# The Comparative Oncology Trials Consortium: Using Spontaneously Occurring Cancers in Dogs to Inform the Cancer Drug Development Pathway

**DOI:** 10.1371/journal.pmed.1000161

**Published:** 2009-10-13

**Authors:** Ira Gordon, Melissa Paoloni, Christina Mazcko, Chand Khanna

**Affiliations:** 1 Radiation Oncology Branch, National Cancer Institute, Bethesda, Maryland, United States of America; 2 Comparative Oncology Program, Center for Cancer Research, National Cancer Institute, Bethesda, Maryland, United States of America

## Abstract

Chand Khanna and colleagues describe the work of the Comparative Oncology Trials Consortium (COTC), which provides infrastructure and resources to integrate naturally occurring dog cancer models into the development of new human cancer drugs, devices, and imaging techniques.

Despite evidence of drug efficacy in mouse models of cancer, many novel anti-cancer agents fail in human cancer patients because of unacceptable toxicity or poor efficacy [1]. Naturally occurring tumors in dogs and other animals have clinical and biological similarities to human cancers that are difficult to replicate in other model systems. A recently launched cooperative effort, the National Cancer Institute's (NCI's) Comparative Oncology Trials Consortium (COTC; http://ccr.cancer.gov/resources/cop/COTC.asp), now provides infrastructure and resources needed to integrate these naturally occurring cancer models into the development of new human cancer drugs, devices, and imaging techniques.

## Disappointments in Cancer Drug Development

Murine cancer models have been extremely useful for analyzing the biology of pathways involved in cancer initiation, promotion, and progression. However, they frequently do not adequately represent many of the features that define cancer in humans, including long periods of latency, genomic instability, and the heterogeneity of both tumor cells and their surrounding microenvironment. Most importantly, the complex biology of cancer recurrence and metastasis, integral to outcomes in human patients, are not appreciably reproduced in the conventional mouse models used in cancer drug development. Furthermore, in many cases, there has been inadequate consideration of relevant exposures for new drugs that are evaluated in mice. The development and approval of novel cancer drugs is lengthy and expensive [2]–[5]; therefore, additional models that better represent the human disease are needed.

Current drug development pathways are frequently unidirectional. Novel agents are assessed in conventional preclinical models of efficacy and toxicity before moving into human clinical trials where they either fail or succeed. Particularly with novel targeted therapies the conventional paradigms of toxicity studies conducted in healthy animals followed by Phase I and Phase II human trials leave unanswered many important questions on the “best use” of these drugs [6]. Translational drug development studies in pet dogs with cancer provide an opportunity to answer these questions by serving as an intermediary between conventional preclinical models and human clinical trials [7]–[9]. In these dogs, cancers develop naturally in the context of an intact immune system and with a syngeneic host and tumor microenvironment. Similar environmental, nutrition, age, sex, and reproductive factors lead to tumor development and progression in human and canine cancers. They share similar features such as histologic appearance, tumor genetics, biological behavior, molecular targets, therapeutic response, and unfortunately, acquired resistance, recurrence, and metastasis.

Clinical trials in pet dogs are not constrained by traditional Phase I, Phase II, and Phase III trial designs. This allows novel agents to be offered to pet dogs before conventional therapies or during the period of minimal residual disease. Pet owners are highly motivated to seek novel options for management of cancer in their pets, especially if conventional treatments do not meet their goals. A pet owner's decision to pursue an investigational treatment is often influenced by the risks associated with this therapy compared to conventional therapy, as well as their expectations for outcomes and reduced costs for care provided by an investigational trial. Additionally, many pet owners are motivated by the opportunity to contribute to the advancement of cancer treatment for future human and canine patients.

The study of cancer biology and therapy in animals with naturally occurring cancers, referred to as comparative oncology, is not a novel concept. Indeed, over the last 30–40 years investigators have used this approach to make important contributions to the understanding and practice of human oncology in fields such as basic tumor biology and immunology [10]–[14], radiation biology [15], hyperthermia [16], and systemic therapies for a variety of cancers including osteosarcoma, lymphoma, melanoma, and others [12],[17]–[22]. One historical limitation to the widespread use and integration of the comparative approach has been a lack of infrastructure to coordinate animal health professionals with the human oncology community, drug developers, and basic scientists.

## Comparative Oncology Trials Consortium Program Infrastructure

The COTC was launched through the intramural NCI's Center for Cancer Research–Comparative Oncology Program. The COTC operates as a collaborative effort between the NCI and extramural academic comparative oncology centers and functions to design and execute clinical trials in dogs with cancer in collaboration with the pharmaceutical industry and nongovernmental groups interested in cancer drug development. Support for the oversight and management of the COTC comes from the NCI. Trial sponsors, most often pharmaceutical companies, support the clinical costs of studies conducted by the COTC academic centers. The goal of this effort is to answer biological questions that can inform the development path of novel agents for future use in human cancer patients in a timely and integrated manner. Trials conducted by the COTC are designed to include clinical and biological endpoints, i.e., pharmacokinetics and pharmacodynamics, so as to optimally inform the design of early phase human trials. Trials are carried out at COTC member institutions, which currently include 18 veterinary academic centers, currently in the United States.

Comparative oncology trials can answer many questions within a single study. The serial collection of tumor and normal tissue biopsies and fluids from the same animal before, during, and after exposure to an investigational agent is feasible. This sequential sampling allows the study of tissue (tumor and/or surrounding normal tissues) endpoints that may be linked to surrogate imaging or circulating biomarkers, as a function of drug exposure or therapeutic response, in ways that are often difficult or unacceptable in human trials. To ensure the integration of such biological endpoints in these studies the COTC Pharmacodynamic (PD) Core was developed (http://ccr.cancer.gov/resources/cop/scientists/pharmacodynamic.asp). The COTC PD Core provides infrastructure to support the development, validation, and assessment of pharmacokinetic, pharmacodynamic, and biological endpoints within COTC trials. Through the COTC and its PD Core, the opportunity now exists to rapidly accrue pet dogs with cancer to clinical trials that are detailed and biologically intensive (http://ccr.cancer.gov/resources/cop/COTC.asp). The first completed consortium trial was recently published [23] and a 12th trial is currently under development. In the interest of open access to this approach and its data, the COTC plans to publish its trials in the journal *PLoS ONE*.

## The Opportunity of the Comparative Approach

Dogs have historically been useful, informative models in the development and discovery of many novel cancer therapeutic strategies. The efficacy of liposomal muramyl tripeptide phosphatidylethanolamine (L-MTP-PE) in dogs with osteosarcoma served as part of the rationale for its evaluation in Phase III studies in children. Indeed, similar results with L-MTP-PE have been observed in both dogs and children [24],[25]. Dogs have been used to develop and evaluate surgical limb sparing techniques [26] and were valuable models in the investigation of the combination of hyperthermia with radiation [27],[28]. Dogs have also been included in the development of novel targeted anticancer agents [20],[29].

The similarities between dog and human cancers are increasingly being realized. The publicly available canine genome has propelled comparative genomics studies. Such studies have shown significant homology between dog and human for recognized cancer-associated genes including *MET*, *IGF1R*, *mTOR*, and *KIT* [9]. Not surprisingly, cytogenetic abnormalities that define human cancers, i.e., *BCR-Abl* translocations in chronic myelogenous leukemia and *RB1* deletions in chronic lymphocytic leukemia have been found in comparable canine cancers [30]. These and other examples have been recently reviewed elsewhere [9].

**Figure 1:**
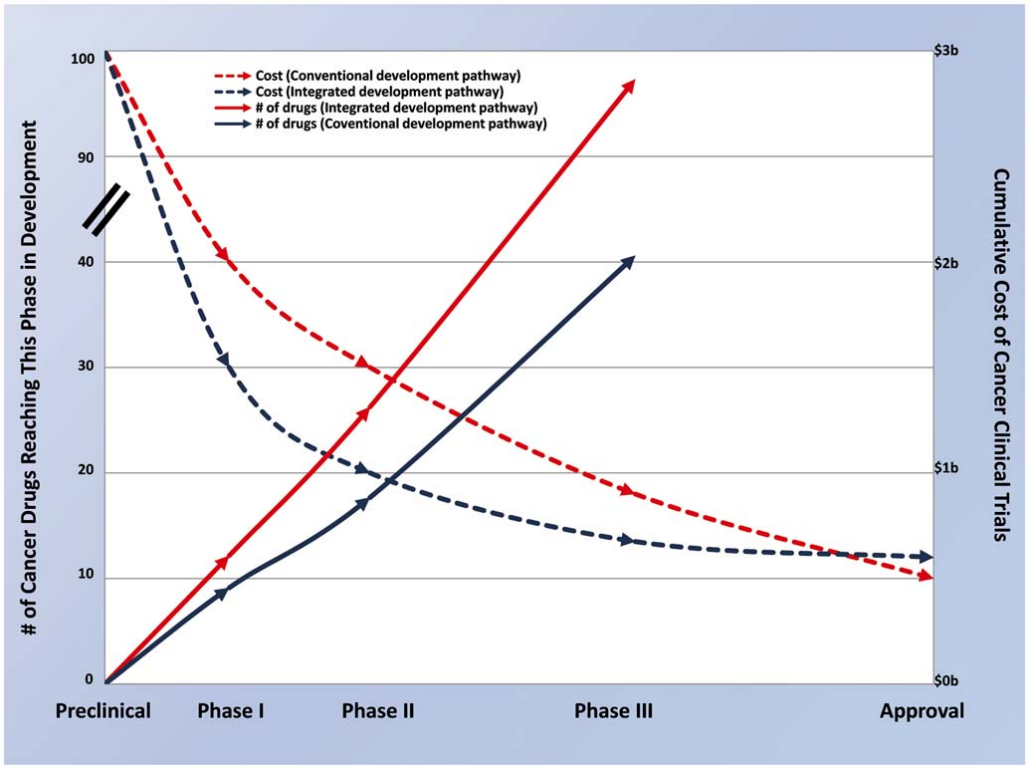
An idealized view of the opportunity provided by a comparative and integrated oncology drug development path. This is a theoretical illustration of 100 preclinical agents that may be evaluated by either a conventional or an integrated and comparative drug development path. Data for transition rates and costs of Phase I, II, and III trials are based on published cost estimates [3] and reported clinical phase transition probabilities for investigational oncology compounds from the 20 largest firms (by pharmaceutical sales in 2005) from 1993 to 2002 [2],[4]. Estimates used to derive a vision of the benefit of an integrated approach to drug development are based, in part, on estimates of transition and approval rates for non-oncology therapeutic areas where informative preclinical models exist [5]. Relative to the conventional development path, the integrated development path is characterized by improved success early in clinical development and a reduction in drug failures late in clinical development. Conventional oncology drug development results in approximately 40% of eligible agents transitioning from preclinical to Phase I, 75% from Phase I to II, 60% from Phase II to III, and 55% from Phase III to approval [2]. Therefore, for every 100 preclinical candidates, only ten new drugs will reach the clinic. Of most significance are failures that occur late in the development path (i.e., after Phase II or Phase III evaluation). With an integrated approach, more toxic and ineffective agents may be eliminated prior to Phase I (estimate 30 agents now entering Phase I trials versus 40 in the conventional pipeline). Attrition in Phase I may be minimized (estimated 87.5% success rate) and an additional 30% of drugs may be removed from development prior to Phase II based on comparative studies that demonstrate poor pharmacokinetics, pharmacodynamics, or activity (estimate 18 agents now entering Phase II trials versus 30 in the conventional pipeline). Deprioritization (from above) of these drugs will improve the Phase II success rate (estimate 90%). Data from comparative studies will result in the removal of 20% of remaining drugs prior to Phase III based on lack of efficacy in the adjuvant setting, thereby improving success in Phase III and leading to 90% of Phase III agents receiving FDA approval (compared to 55% in the conventional pipeline). In this model, 12 new drugs out of every 100 preclinical candidates will reach the clinic. Using estimates for Phase I, II, and III trials of US$15.2 million, US$23.5 million, and US$86.3 million per trial respectively [3], the total clinical trial expenditures for developing 100 preclinical agents is US$2.87 billion using conventional methods. Using the hypothetical improvements described above that result from the integrated approach the clinical costs for development will be US$2.03 billion [3]. Factoring in additional costs for comparative studies with this approach of US$150,000 for studies conducted in the preclinical setting, US$250,000 for studies conducted before or during Phases I–II human trials and US$1 million for studies conducted before Phases II–III studies, the total cost of development is estimated at US$2.07 billion. The result may be a decrease in average clinical trial costs per approved drug from US$290 million to US$173 million [5].

Integrating the comparative approach has the opportunity to improve the development path of new cancer drugs (Figure 1). Drugs that may be less likely to succeed in early human clinical trials may be identified and culled early. For example, the addition of comparative oncology studies in the preclinical setting will eliminate drugs with an unfavorable therapeutic index or inferior target modulation attributes, thus identifying agents most likely to succeed in human Phase I trials. Comparative studies performed during or after human Phase I studies may focus on pharmacokinetic/pharmacodynamic endpoints, classify responding patient subsets, and identify optimal drug combinations. These data may eliminate inactive drugs before Phase II human trials and optimize the design of these trials. Furthermore, the integration of studies using pet dogs with cancer provides a unique opportunity to assess efficacy in the adjuvant or minimal residual disease setting and, in so doing, may prioritize those agents most likely to be effective as Phase III human cancer agents. Collectively, the elimination of inferior drugs early in development will reduce drug attrition in later phases of human clinical development and result in fewer human participants entering trials with potentially ineffective or unsafe drugs. By reducing the number of drugs entering each phase of drug development and increasing the success rate in Phase III trials, an integrated approach can substantially decrease the costs and risks of drug development (Figure 1).

## Challenges and Limitations

As with all novel approaches and perspectives, integrating studies with pet dogs with cancer into the development pathway is associated with some hesitation and perception of risk. One of the goals of the COTC is to define and address perceived risks and actual challenges and to mitigate them when possible.

- **Timelines** for the completion of a study in pet dogs are longer than those in rodent models. The multicenter consortium that makes up the COTC was developed to address this issue. By integrating these studies into the development pathway, human and pet dog studies can be performed to strategically prevent delays in the conduct or completion of human clinical trials.
- **Reporting of data** in a timely matter is an important aspect of a clinical trial. The COTC has developed an electronic reporting system to acquire data in real time and provide oversight and monitoring of study results.
- **Oversight guidelines** of these types of trials in pet dogs are not yet fully defined. In all cases, the care of pet animals must be given great consideration and include institutional Animal Care and Use Committee (ACUC) approval. Trials are conducted in a manner that prioritizes the medical care and health of animals and requires written owner consent. Reasonable procedures for a given study are assessed on a case by case basis and may be overseen by a Data Safety Management Board that functions with the ACUC. A working guide to the conduct and regulatory reporting of comparative trials is currently in development.
- **Study cost** and budget must be considered in the assessment of this approach. The relatively larger size of dogs mandates a concomitantly larger drug supply for these trials than do traditional murine studies. However, preclinical trials of novel human cancer drugs do not require good manufacturing practice for drug use. Tumor-bearing dog studies are more expensive than mouse studies but are within range of other large animal toxicity studies necessitated for Investigational New Drug application. The costs for these studies depend on the trial design, which varies based on the specific questions asked and answered. Entry criteria and endpoints vary, but most studies are powered similarly to corresponding human Phase I/II trials based on the statistical considerations for the questions to be answered. The addition of serial tumor biopsies, imaging, or other correlative endpoints incrementally add to study costs, but these additions add value to the drug development pathway previously not recognized. If an integrated approach is successful at prioritizing drugs in development and optimizing human clinical trials, these study costs will be minor compared to the substantial reduction in costs seen in human clinical trials (Figure 1).
- **Comparable histology** is not always available in the comparative approach. In dogs, the most common tumors are sarcomas and lymphoid neoplasms, whereas some of the common cancers of humans, namely breast, prostate, gastrointestinal, and lung carcinomas, are less common in dogs. Clinical studies of these cancers in dogs may need more time for completion or addition of broader, potentially international clinical trial centers to enhance patient accrual. In the future, it is likely that cancer therapeutics will not be defined by their activity within a particular histology, but instead by a specific cancer biology or dysregulation of a pathway or gene. As such, a focus on common histology might be replaced by one on genetic or molecular similarities.
- **Common targets** for a therapy may not always be readily known or available for human and dog tumors. Humanized antibodies and proteins may not interact identically in dogs or may be inactivated by their immune system. In some cases, the dose intensity of a drug, as used in humans, may cause unacceptable toxicities in dogs. For example, dogs are particularly sensitive to the cremophor vehicle used for paclitaxel. This has largely limited the evaluation of conventional taxanes in dogs.

## The Future of the COTC and the Comparative Approach

The increasing availability of banked canine tumors and associated “omic” annotations for these cancers will allow for rapid identification of valid tumor targets in canine cancers. To this end, a second community initiative, the Canine Comparative Oncology and Genomics Consortium (CCOGC; http://www.ccogc.net/) was recently developed to facilitate strategic partnerships and collaborations across a diversity of these disciplines and to develop a tissue biospecimen repository. This repository has initiated sample collections and expects to provide tissues to the community in late 2009.

Proceeding forward, the COTC plans to increase awareness of the applications of the comparative approach. Through this effort a greater understanding about the diseases and treatment agents that are best suited to this approach will be developed; a broader integration of this approach into the drug development and approval process is expected to emerge; and an acceleration of the development of effective new anticancer agents, devices, and imaging techniques will occur. We believe that such efforts will advance the quality of care for both human and veterinary cancer patients.

## Abbreviations

ACUC: Animal Care and Use Committee
COTC: Comparative Oncology Trials Consortium
NCI: National Cancer Institute
